# Regulatory T cells participate in the recovery of ischemic stroke patients

**DOI:** 10.1186/s12883-020-01648-w

**Published:** 2020-02-28

**Authors:** María Santamaría-Cadavid, Emilio Rodríguez-Castro, Manuel Rodríguez-Yáñez, Susana Arias-Rivas, Iria López-Dequidt, María Pérez-Mato, Manuel Rodríguez-Pérez, Ignacio López-Loureiro, Pablo Hervella, Francisco Campos, José Castillo, Ramón Iglesias-Rey, Tomás Sobrino

**Affiliations:** Clinical Neurosciences Research Laboratory, Clinical University Hospital, Health Research Institute of Santiago de Compostela (IDIS), Hospital Clínico, c/ Travesa da Choupana, s/n, 15706 Santiago de Compostela, Spain

**Keywords:** Early neurological deterioration, Interleukin-10, Ischemic stroke, Neuroinflammation, Regulatory T cells, Risk factors

## Abstract

**Background:**

Recent preclinical studies have shown that regulatory T cells (Treg) play a key role in the immune response after ischemic stroke (IS). However, the role of Treg in human acute IS has been poorly investigated. Our aim was to study the relationship between circulating Treg and outcome in human IS patients.

**Methods:**

A total of 204 IS patients and 22 control subjects were recruited. The main study variable was good functional outcome at 3 months (modified Rankin scale ≤2) considering infarct volume, Early Neurological Deterioration (END) and risk of infections as secondary variables. The percentage of circulating Treg was measured at admission, 48, 72 h and at day 7 after stroke onset.

**Results:**

Circulating Treg levels were higher in IS patients compared to control subjects. Treg at 48 h were independently associated with good functional outcome (OR, 3.5; CI: 1.9–7.8) after adjusting by confounding factors. Patients with lower Treg at 48 h showed higher frequency of END and risk of infections. In addition, a negative correlation was found between circulating Treg at 48 h (*r* = − 0.414) and 72 h (*r* = − 0.418) and infarct volume.

**Conclusions:**

These findings suggest that Treg may participate in the recovery of IS patients. Therefore, Treg may be considered a potential therapeutic target in acute ischemic stroke.

## Background

Stroke represents the second cause of death in Europe and developed countries. In addition to mortality, long-term morbidity remains as one of the main consequence associated to stroke patients [1, 2]. Although stroke causes enormous medical and economic costs associated to stroke, thrombolysis with recombinant plasminogen activator (rtPA) remains as the only approved pharmacological treatment for ischemic stroke (IS). However, there is a narrow therapeutic window for the use of rtPA treatment (< 4.5 h) due to side effects such as hemorrhagic transformation or treatment with previous anticoagulants [3]. Because of these limitations, rtPA is only available for a small percentage of IS patients in developed countries, being this situation worst in developing countries. Therefore, new and effective therapies are highly demanded in clinical practice.

Stroke triggers an acute immunological and inflammatory response in the brain that participates actively in the evolution of ischemic damage. In the last years, the interest in the role of inflammation in stroke pathogenesis has significantly increased, becoming an important target for future therapeutic drugs. In this regard, it is well known that decrease of blood flow in a brain area causes neuronal necrosis and leads to an immune response and invasion of inflammatory cells in the ischemic tissue, which mediates a secondary brain injury [4–6]. However, although immune response contributes to brain tissue damage, therapeutic strategies based on immunosuppression have failed to show efficacy in clinical trials [7].

Alternatively to the use of immunosuppressant drugs, other mechanisms involved in the control and regulation of inflammatory response have been recently proposed aimed to prevent brain damage after stroke. In this regard, regulatory T cells (Treg) are a subgroup of CD4 T lymphocytes that play an important role in maintaining immune homeostasis, preventing autoimmunity and inflammation. Due to their immunomodulatory function, it has been proposed that Treg may play an important role in the pathophysiology of IS [8].

Several preclinical studies have tested the therapeutic role of Treg in cerebral ischemia, finding that their depletion causes larger infarct volumes [9], while their exogenous administration mediates a protective effect [10]. However, few clinical studies have investigated the role of Treg in IS patients [11–16]. These studies showed controversial results, probably due to the small and heterogenous group of patients included in the studies. Moreover, those clinical trials did not evaluate the relationship between circulating Treg levels with infarct volume, patient’s outcome or early neurological deterioration (END). In addition, there is a lack of studies investigating the temporal profile of Treg during acute phase of IS. Similarly, there is no data about the association of Treg and interleukin-10 (IL-10), a cytokine with anti-inflammatory properties, which is considered as the main effector mechanisms of Treg [17]. These data could help to establish the potential use of Treg as a therapeutic target able to improve functional outcome in IS patients.

In this clinical study, we have analyzed the association between circulating levels of Treg with the functional outcome in IS patients. Likewise, it was analyzed if higher levels of Treg are related with smaller infarct volumes and less frequency of END. Finally, we have studied the correlation between circulating Treg and serum levels of IL-10.

## Methods

### Patient’s characteristics

Ischemic stroke patients within 12 h from symptoms onset were prospectively included in the study between April 2013–July 2014. A cohort of control subjects matched by sex and age was included.

Inclusion criteria were: hospitalized patients with first-episode of IS within 12 h from symptoms onset; age > 18 years; previously independent for their daily living activities (modified Rankin Scale (mRS) ≤1).

Exclusion criteria were: presence of intracerebral hemorrhage confirmed by neuroimaging; previous IS; cancer or severe systemic disease that determine a life expectancy lower than 6 months; infections during the last 30 days before admission; chronic inflammatory disease; pregnancy; renal replacement therapy; treatment with steroids, immunosuppressive and immunomodulatory drugs or antibiotics during the last 30 days before admission; periodontal disease; and fever in the previous 72 h (axillary temperature over 38 °C). Patients with active infection (axillary temperature > 37.5 °C and leukocyte levels > 15,000/μL or < 4000/μL), cough and spitting, voiding dysfunction, diarrhea and clinical signs of endocarditis or meningitis.

On the other hand, a cohort of subjects without any neurological, inflammatory or infectious disease was included as control group. The selection of these control subjects was made by inviting the patient’s relatives to participate in the study. Control subjects were matched to patients by gender and age.

### Clinical variables and neuroimaging studies

All patients were admitted in the Stroke Unit of University Clinical Hospital of Santiago de Compostela and treated according to the guidelines of the Cerebrovascular Diseases Study Group of the Spanish Society of Neurology [18]. Medical history recording demographic data, potential vascular risk factors, blood counts, biochemistry and coagulation tests, 12-lead ECG, chest radiography, carotid and transcranial ultrasonography and Computed Tomography (CT) or Magnetic Resonance Imaging (MRI) were performed at admission.

To evaluate neurologic deficit, the National Institute of Health Stroke Scale (NIHSS) was performed at admission, 24, 48 and 72 h, at discharge, and at 3 months. END was defined as an increase of 4 points or more in NIHSS assessment between baseline and any other NIHSS evaluation during the first 72 h. Functional outcome was evaluated at discharge and at 3 months by mRS. NIHSS and mRS were evaluated by internationally certified neurologists. Stroke etiology was classified according to TOAST criteria [19].

We evaluated the incidence of any infection during the hospitalization period. A protocol has been implemented in order to evaluate the presence of infections during the acute phase of stroke. The following tests were performed in those patients who showed an axillar temperature > 37.5 °C in two different determinations separated by 1 h, or in patients with one axillar temperature determination > 38 °C: blood counts, biochemistry analysis and blood culture; physicians made a clinical suspicion regarding the infection origin. During the etiological examination of the infection origin, empiric antibiotherapy was started according to clinical suspicion. Once the antibiogram was obtained, specific antibiotic treatment was started in case of positive cultures.

To evaluate infarct volume, a control CT was performed between 4th–7th days after IS. Infarct volume was quantified in cubic centimeters (cm^3^) and was assessed according to the formula 0.5xAxBxC, where A and B correspond to higher diameters in perpendicular direction and C to the number of 10 mm slices where infarct volume is present [20]. All neuroimaging evaluations were made by the same neuroradiologist blinded to clinical and laboratory data.

### Quantification of Treg

Circulating levels of Treg were measured by flow cytometry according to methods and using the markers described elsewhere [21–23]. Prior to patient’s inclusion, we selected 20 IS patients who matched inclusion/exclusion criteria to evaluate the optimal temporal profile for the quantification of Treg during the acute phase of IS. Blood samples were collected with an evacuated tube system (Vacutainer) in EDTA tubes at baseline, 24, 48, 72 h and at days 4, 5 and 7. Based on to this temporal profile, we obtained blood samples in the more relevant time-points for Treg evaluation (i.e. at admission, 48 and 72 h and 7th day).

Blood samples were processed within 3 h after collection by a single researcher who was blinded to patients’ clinical, biochemical or radiological results. Circulating Treg were analyzed for the expression of specific surface antigens with direct flow cytometry (BD FACSAria IIu, BD, Franklin Lakes, NJ, USA). In brief, 50 μL of peripheral blood were labelled with 10 μL of FITC-conjugated anti-CD4 (BD, Franklin Lakes, NJ, USA), 10 μL of PE-conjugated anti-CD25 (BD, Franklin Lakes, NJ, USA), and 10 μL of Alexa Fluor® 647-conjugated anti-CD127 (BD, Franklin Lakes, NJ, USA) monoclonal antibodies. We considered Treg as CD4+/CD25+/CD127- staining cells in the lymphocyte gate. In all analyses, 2.5 × 10^5^ events were acquired, using a FACSAria IIu analyzer (BD, Franklin Lakes, NJ, USA), and processed using the PC FACSDiva software program (BD, Franklin Lakes, NJ, USA). Treg count was expressed as percentage of Treg over total analyzed lymphocytes.

### IL-10 determination

Blood samples, drawn from all patients at admission, and at 24 ± 6, 48 ± 12, and 72 ± 12 h, were collected in glass chemistry test tubes, centrifuged at 3000 rpm during 10 min, and immediately frozen and stored at − 80 °C. Serum levels of IL-10 were measured using an immunodiagnostic IMMULITE 1000 System (Siemens Healthcare España, Madrid, Spain). Determinations were performed in an independent laboratory blinded to clinical and neuroimaging data.

### Outcome variables

The primary endpoint was good functional outcome (mRS ≤2) at 3 months. Infarct volume and presence of END were evaluated as secondary outcome variables. The development of infections during hospitalization was recorded as safety variable. Finally, we analyzed the correlation between circulating Treg and serum levels of IL-10 in order to investigate the possible mechanism of action of Treg.

### Statistical analysis

Sample size was calculated using the statistical EPIDAT 3.1 software, considering that those patients within the highest quartile regarding Treg levels during the first week after stroke achieve a 25% more frequency of good outcome at 3 months compared with those with Treg levels in the lowest quartile. The minimum calculated sample size was 172 patients in order to obtain a statistical power of 80% with a significant difference level of 0.05.

Results were expressed as percentages for categorical variables and as mean (SD) or median and range (25th and 75th percentiles) for the continuous variables depending on whether their distribution was normal or not. The Kolmogorov-Smirnov test was used for testing the normality of the distribution. Proportions were compared using the chi-square or Fisher test, while the continuous variables between groups were compared with the Student’s t or the Mann-Whitney tests depending on whether their distribution was normal or not, respectively. In case of more than 3 groups, variables were compared using ANOVA test. Bivariate correlations were performed using Pearson’s (normally distributed variables) or Spearman (variables without normal distribution) coefficients.

ROC curves were used to establish the best cut-off point for Treg levels that optimally predicted good functional outcome.

The independent association of circulating Treg levels with good functional outcome at 3 months and the risk of infections was assessed by logistic regression analysis; while their independent influence on infarct volume was assessed by multiple linear regression models. Each logistic regression analysis or multivariable linear regression model was adjusted for those significant variables in the bivariate analysis. Residual plots were examined to detect potential non-linear relationships between the outcome variable and continuous independent variables. Results were expressed as adjusted odds ratios (ORs) or Beta estimate with the corresponding 95% confidence intervals (95% CI). A *p*-value < 0.05 was considered to be statistically significant in all tests. The statistical analysis was conducted in SPSS 20.0 (IBM, Chicago, IL, USA) for Mac.

## Results

Twenty-two control subjects were included. No differences were found between controls and IS patients regarding age, sex, previous history of hypertension, diabetes, dyslipidemia, coronary disease, peripheral artery disease, and alcohol or tobacco consumption. IS patients showed more prevalence of atrial fibrillation than control subjects (42.2 vs. 0%, *p* < 0.0001).

On the other hand, 335 IS patients admitted in the Stroke Unit within the first 12 h from stroke onset were evaluated to be included in the study. Among those, 204 patients fulfilled all inclusion criteria and did not fulfill any exclusion criteria. One hundred three patients (50.5%) were males. Mean age was 71.7 ± 10.6 years. The NIHSS score at admission was 8[4, 12] and the mean infarct volume was 50.8 ± 88.4 cm^3^. Regarding stroke etiology, we found 93-cardioembolic (45.6%), 26-atherothrombotic (12.7%), 7-lacunar (3.4%) and 78-undetermined (38.2%).

Circulating levels of Treg (0.0222 ± 0.0177 vs. 0.0013 ± 0.0009%; *p* < 0.0001) as well as IL-10 serum levels (6.9 ± 1.7 vs. 1.8 ± 0.1 pg/mL; *p* < 0.0001) at admission were higher in IS patients than in control subjects.

**Fig. 1:**
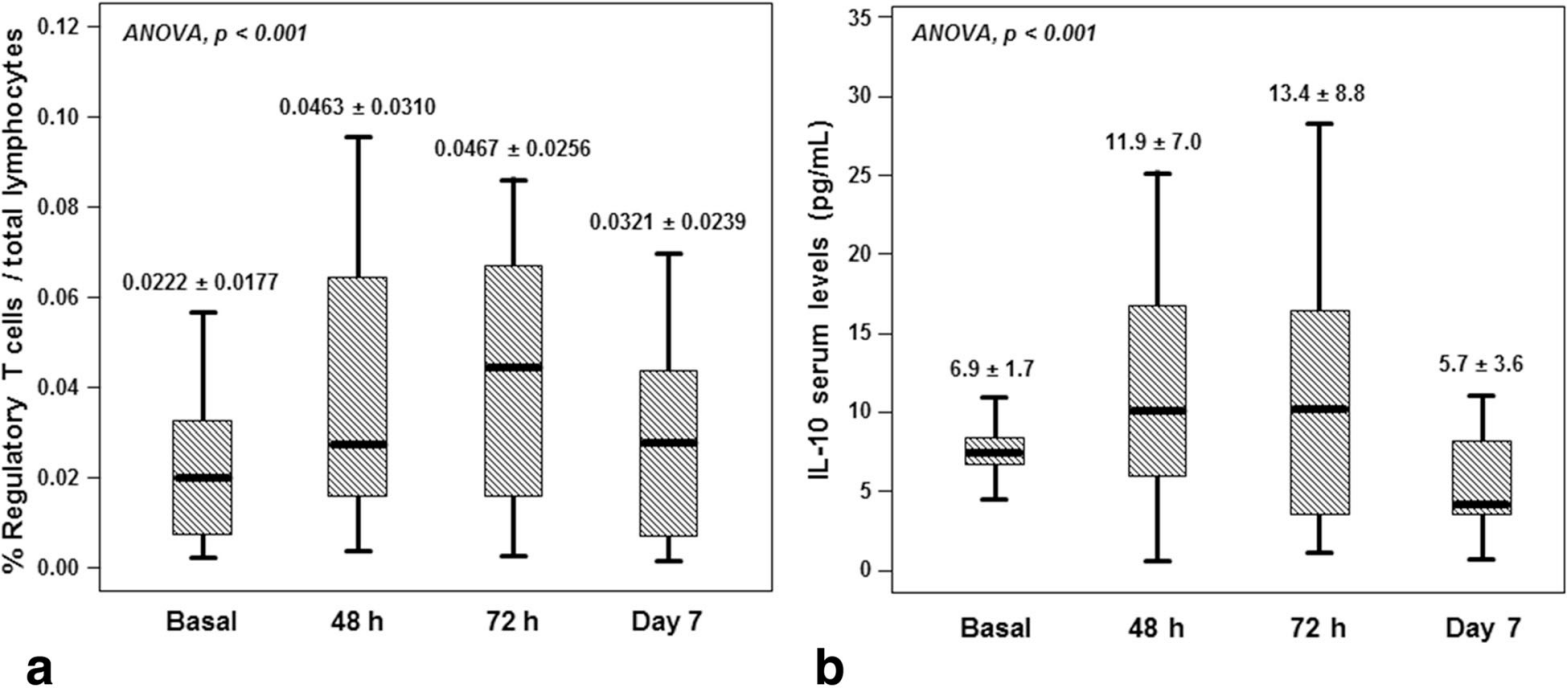
Temporal pattern of % Treg (**a**) and IL-10 levels (pg/mL) (**b**) at admission, 48 and 72 h and day 7

The temporal pattern of Treg and IL-10 levels are shown in Fig. 1. We found that circulating Treg were significantly higher at 48, 72 h and day 7 in relation to the baseline measurement. Based on these results, we evaluated this temporal profile in the complete cohort of IS patients included in the study.

### Primary endpoint: influence of Treg on functional outcome

**Fig. 2:**
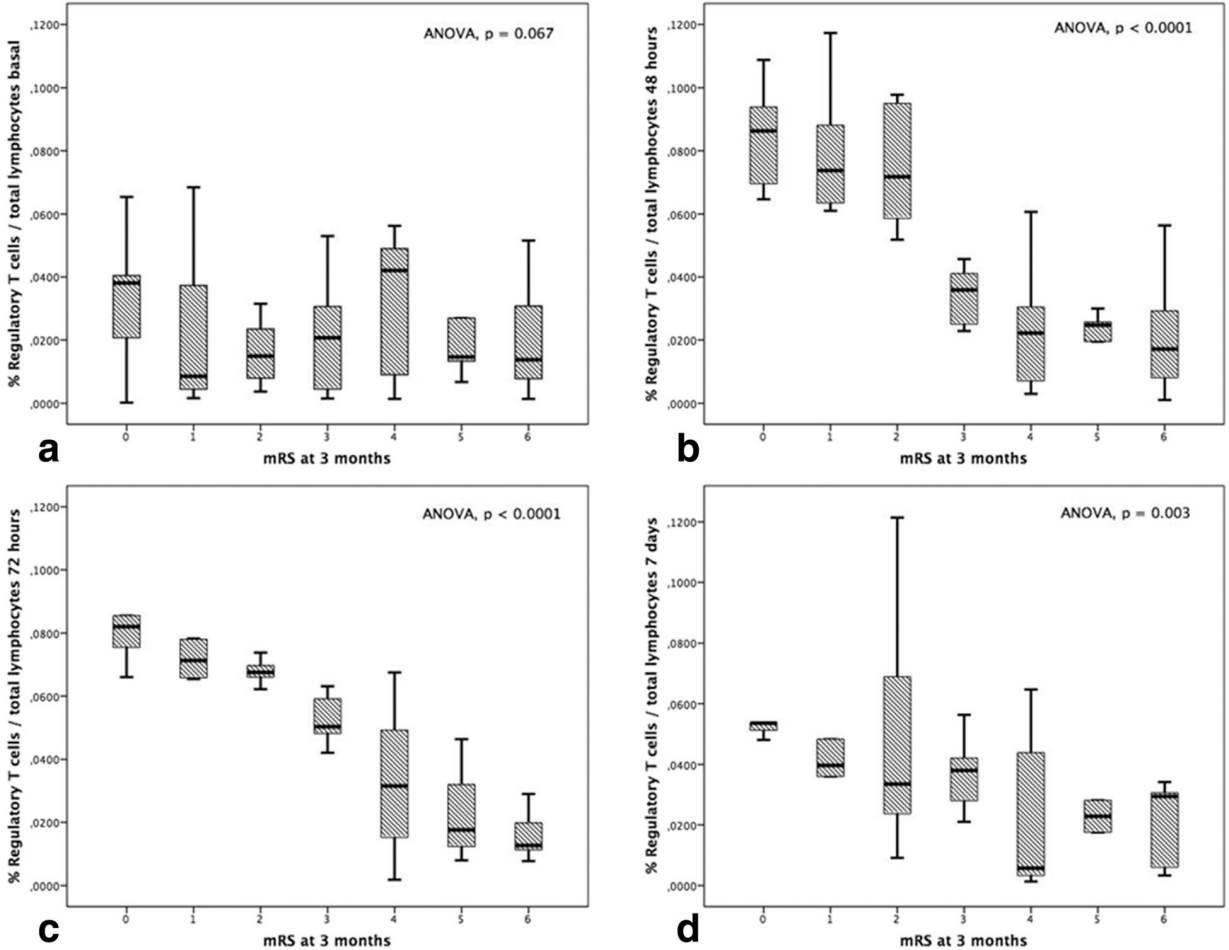
Association between mRS score at 3 months and circulating levels of Treg at admission (**a**), 48 h (**b**), 72 h (**c**) and day 7 (**d**)

Patients with lower mRS scores at 3 months showed higher levels of Treg at 48, 72 h as well as at day 7, but not at admission (Fig. 2). Table 1 shows the baseline clinical characteristics, vascular risk factors, stroke subtype, biochemical/cellular parameters and Treg levels of patients with good (*n* = 87; 36.2%) and poor outcome (*n* = 117; 63.8%) at 3 months. We found that patients with good outcome had higher levels of Treg at 48 h (*p* < 0.0001), 72 h (*p* < 0.0001) and 7 days (*p* = 0.001), but not at admission (*p* = 0.962).

**Table 1:** Baseline clinical characteristics, vascular risk factors, stroke subtype, biochemical/cellular parameters and neuroimaging findings in patients with good or poor outcome at 3 months

Variable	Good outcome *n* = 87	Poor outcome *n* = 117	*P* value
Age (years)	57.4 ± 8.4	76.3 ± 7.3	< 0.0001
Female gender, n (%)	36(42.7)	67(57.3)	0.548
Previous hypertension, n (%)	32(36.9)	74(63.2)	0.030
Previous diabetes, n (%)	31(36.2)	75(64.1)	0.197
Previous dyslipidemia, n (%)	28(32.5)	79(67.5)	0.016
Previous atrial fibrillation, n (%)	40(46.7)	63(53.8)	< 0.0001
Previous ischemic cardiopathy, n (%)	65(75.0)	29(24.7)	0.059
Previous peripheric arteriopathy, n (%)	0(0)	100 (100)	0.220
Alcohol consumption, n (%)	39(45.5)	64(54.7)	0.475
Smoking, n (%)	47(54.5)	53(45.3)	0.052
Previous statin consumption, n (%)	33(37.9)	73(62.4)	0.184
Leukocyte at admission (× 10^3^/mmc)	8.1 ± 1.7	8.7 ± 3.8	< 0.0001
Glucose at admission (mg/dL)	132.5 ± 39.3	155.8 ± 89.2	< 0.0001
Fibrinogen at admission (mg/dL)	380.0 ± 64.9	400.4 ± 101.8	< 0.0001
CRP(C reactive protein) (mg/L)	1.5 ± 1.7	3.2 ± 4.8	< 0.0001
Recanalization therapy, n (%)	55.6	44.4	0.433
Basal NIHSS	4 [2, 7]	13 [7, 18]	< 0.0001
TOAST			0.003
- Cardioembolic, n (%)	27 (31.0)	66 (56.4)	
- Aterothrombotic, n (%)	13 (14.9)	13 (11.1)	
- Lacunar, n (%)	3 (3.4)	4 (3.4)	
- Undetermined, n (%)	44 (56.4)	34 (43.5)	
% Treg / total lymphocytes at admission	0.0224 ± 0.0177	0.0197 ± 0.0112	0.962
% Treg / total lymphocytes, 48 h	0.0715 ± 0.0133	0.0231 ± 0.0173	< 0.0001
% Treg / total lymphocytes, 72 h	0.0709 ± 0.0064	0.0273 ± 0.0183	< 0.0001
% Treg / total lymphocytes, 7 days	0.0515 ± 0.0189	0.0209 ± 0.0172	0.001

ROC analysis showed that Treg levels at 48 h ≥ 0.0550% suggested good functional outcome 3 months with a specificity of 97% and a sensitivity of 95% (area under the curve: 0.990; 95% CI: 0.997–1.000; *p* < 0.0001). Similarly, Treg levels at 72 h ≥ 0.0650% suggested good outcome at 3 months with a specificity of 95% and a sensitivity of 93% (area under the curve: 0.964; 95% CI: 0.886–1.000; *p* < 0.0001).

In the logistic regression analysis, Treg levels at 48 h were independently associated with good functional outcome at 3 months (OR 3.5; 95% CI: 1.9–7.8; *p* < 0.0001) after adjustment by age, previous history of hypertension, dyslipemia, atrial fibrillation, leukocyte counts, glucose and fibrinogen levels, high- sensitive C-reactive protein levels, basal NIHSS and cardioembolic stroke. Treg levels at 72 h were independently associated with good functional outcome at 3 months (OR 1.7; 95% CI: 1.1–3.1; *p* = 0.016) after adjustment by the same variables.

### Early neurological deterioration (END)

END was observed in 13 patients (6.4%). Circulating Treg levels at 48 h (0.0132 ± 0.0125% vs. 0.0411 ± 0.026%; *p* < 0.0001) and 72 h (0.0096 ± 0.0061% vs. 0.0453 ± 0.0234%; *p* < 0.0001) were lower in patients who suffered END, however, due to the small number of patients with END it was not possible to perform a logistic regression analysis.

### Infarct volume

Infarct volume was measured in 195 patients. We found a negative correlation between infarct volume and circulating levels of Treg at 48 h (*r* = − 0.414; *p* < 0.0001) and 72 h (*r* = − 0.418; *p* < 0.0001). No correlation has been found between infarct volume and Treg levels at baseline and at day 7.

In the multivariate analysis, Treg levels at 72 h (B: -648.9; 95% CI: − 1251.2 to − 46.8; *p* = 0.035), but not at 48 h (B: -545.4; 95% CI: − 1036.5 to 291.6; *p* = 0.199) were independently associated with the infarct volume after adjustment by age, previous history of hypertension, dyslipemia, atrial fibrillation, leukocyte counts, glucose and fibrinogen levels, high-sensitive C-reactive protein levels, basal NIHSS and cardioembolic stroke.

### Risk of infections

Twenty-six patients (12.7%) developed infections during the hospitalization period: 17 (65.4%) had respiratory infections, 6 (23.1%) urinary infections and in 3 patients (11.5%) the origin was unknown. Infection during hospitalization was associated with higher temperature at 24 h (38.1 ± 0.3 °C vs. 36.6 ± 0.5 °C; *p* < 0.0001) and at 48 h (37.9 ± 0.5 °C vs. 36.7 ± 0.5 °C; *p* < 0.0001), and with a greater neurological deficit at admission (NIHSS 14[11, 20] vs. 9[5, 18]; *p* = 0.033). The presence of infections during the hospitalization was associated with poor functional outcome at 3 months; patients with infection showed higher scores of mRS at 3 months (5 [4, 6] vs. 3 [1, 4]; *p* < 0.0001).

Circulating Treg levels at 48 h were lower in patients with infections (0.0189 ± 0.009% vs. 0.0425 ± 0.0280%; *p* < 0.0001). Similar results were found for Treg levels at 72 h (0.0168 ± 0.0105% vs. 0.0473 ± 0.0238%; *p* < 0.0001).

In the logistic regression analysis, lower Treg levels at 48 h (OR: 0.35; 95% CI: 0.00–0.57; *p* = 0.001) and 72 h (OR: 0.24; 95% CI: 0.02–0.51; *p* < 0.0001) were independently associated with infections during hospitalization after adjusting by age, previous history of hypertension, dyslipemia, atrial fibrillation, leukocyte counts, glucose and fibrinogen levels, high-sensitive C-reactive protein levels, basal NIHSS and cardioembolic stroke.

### Circulating Treg and serum levels of IL-10

**Fig. 3:**
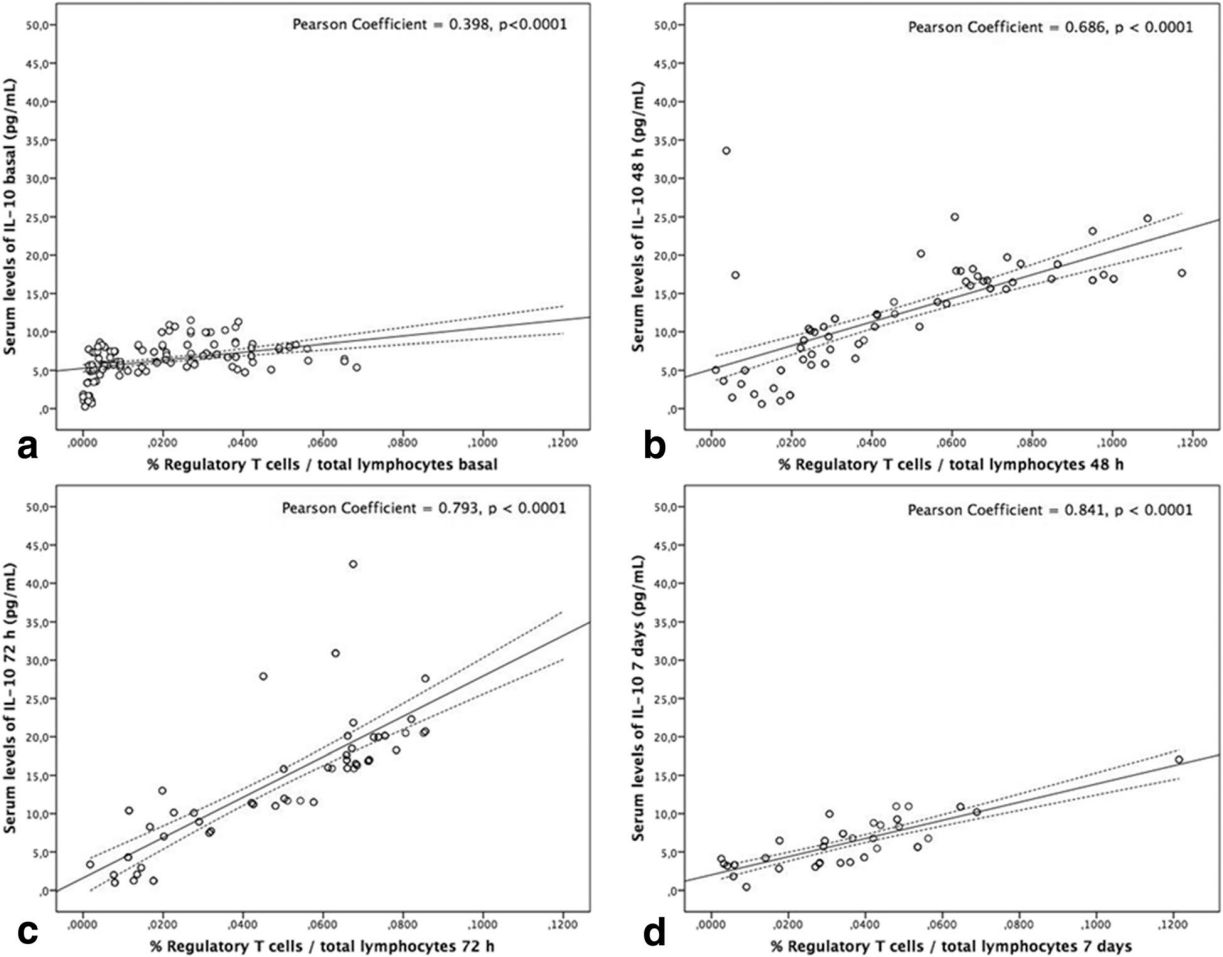
Association between circulating Treg and serum levels of IL-10 at admission (**a**), 48 h (**b**), 72 h (**c**) and day 7 (**d**)

We found a positive correlation between Treg and IL-10 levels for the 4 time points analyzed. However, this association was stronger the later after stroke during the first 7 days (Fig. 3).

## Discussion

This study evaluated the relationship between circulating levels of Treg (defined as CD4+/CD25+/CD127) and brain injury in IS patients. Circulating Treg levels at 48 and 72 h were independently associated with good functional outcome at 3 months. This favourable effect on the primary endpoint was supported by positive effects observed on the infarct volume, END and reduction of infections during hospitalization.

The results showed higher levels of IL-10 in patients with ischemic stroke compared with controls. A previous study comparing IL-10 serum levels between stroke patients and healthy population [24] reported contrary results, finding lower levels of IL-10 in stroke patients compared to controls. In this research work, it is noted that small number of patients were included (42 stroke patients and 39 healthy control subjects), in which the mean age of these patients is about 10 years younger than the patients included in our study. In addition, different exclusion criteria were used as active infection-defining temperature or leukocyte levels. Our results suggest that levels of Treg and IL-10 increase during the acute phase, and could exert a pathophysiological role in IS.

Previous studies in animal models of cerebral ischemia showed an increase of Treg infiltration brain tissue at days 14 and 30 after middle cerebral artery occlusion (MCAO) [25]. In our study we used peripheral blood samples to determinate the temporal pattern of Treg levels during the acute phase of IS. We found that circulating levels of Treg increase during the first 3 days from stroke onset, showing a subsequent but not significant decrease at day 7. Therefore, our results represent a clinical association of the potential role of Treg during the first phase of acute IS. Our results differ from those found in previous small (46 stroke patients and 12 healthy control subjects) and heterogeneous clinical studies (ischemic and hemorrhagic stroke patients were included) that described a decrease in circulating Treg levels at the second day after stroke, followed by a significant increase at day 7 [16].

We found that higher levels of Treg during the acute phase of IS were independently associated with good functional outcome at 3 months. Our results disagree to those reported by Urra et al. [16] that did not find a relationship between Treg levels and functional outcome of IS patients. The difference could be explained by the small sample size limitation evaluated, the inclusion of ischemic and hemorrhagic stroke patients in that study, and the exclusion criteria used. To the best of our knowledge, this is the first prospective study that specifically analyzed the association between circulating Treg during the acute phase of IS and long-term outcome.

Our results showed that patients with END had lower levels of Treg during the acute phase of stroke. The sample size of patients who suffered END was not enough to perform a multivariate analysis to determinate whether the effect of Treg on END could be a direct cause or if it acts as a surrogate marker. Lower IL-10 levels were associated with clinical worsening [26], but we did not find previous studies analyzing the relationship between END and Treg, so this aspect should be investigated in further studies.

We also studied the association between Treg levels and infarct volume, since it has not been previously reported in literature. We found that higher levels of Treg were related with smaller infarct volume. These results also suggest a potential beneficial role of Treg in acute IS, probably by decreasing inflammation which is reflected in a reduction of infarct volume.

Several mechanisms have been proposed for Treg in stroke [27, 28] such as the production of anti-inflammatory cytokines, elimination through granzymes and perforins and metabolic mechanisms. In the context of experimental cerebral ischemia, several studies have demonstrated that IL-10 is a key neuroprotective cytokine involved in the regulation of post-stroke neuroinflammation [9, 29]. In the brain, Treg together with B regulatory (Breg) cells and microglial/monocytes represent the main sources of IL-10. Previous studies in animal models of cerebral ischemia have confirmed the role of IL-10 as a mediator of the protective effect mediated by Treg [29, 30]. In fact, preclinical strategies directed towards the increase of lymphocyte IL-10 production [30, 31] or exogenous IL-10 administration have shown to improve outcome [29]. Therefore, we studied the relationship between Treg and IL-10 levels in IS patients. We found a correlation between IL-10 and Treg levels at admission, 48 and 72 h, and day 7, supporting the possible anti-inflammatory role of Treg by increasing IL-10 levels, demonstrated in preclinical studies [29, 30]. Previous clinical studies have established a positive association between higher IL-10 levels during the acute phase and good outcome in IS [26, 32–36]. Nevertheless, no studies had previously investigated the role of IL-10 as a possible mediator of Treg in acute phase of IS in humans.

Other objective of this study was to evaluate the effect of Treg on the risk of systemic infections. Systemic infections are frequent complications during the acute phase of stroke (7–35%, depending on the series) [37], and its presence worsens the long-term outcome [37–43]. Some authors proposed that stroke may induce a systemic immunosuppression that could increase the risk of infections [39]. However, the underlying mechanisms that result in widespread immunosuppression after stroke and subsequent systemic infections are unknown. A study has observed lymphopenia and increased apoptosis of Th lymphocytes, cytotoxic T lymphocytes and B lymphocytes at early phases of stroke [37]. Increased levels of cortisol and metanephrine have been also related with the risk of infections after stroke [44, 45]. It has been described that during the first hours after stroke, pro-inflammatory cytokines are up-regulated (IL-6, IL-1, TNFα, IL-8, MCP-1, etc) [46]. This inflammation stimulates both the hypothalamic-pituitary-adrenal axis and sympathetic nervous system, which suppresses immune cell function and can be related to systemic downregulation of the immune system [37]. On the other hand, a manuscript by Hug et al. [47] has already described the impact of stroke volume in a clinical cohort on lymphocyte counts. This study identified infarct volume as a major determiner of the extent of post-ischemic lymphocytopenia and monocyte dysfunction, which are markers of susceptibility to infection.

Treg are a subpopulation of cells with immunosuppressive effects [48, 49], so these cells could be related with the risk of infections during the acute phase of stroke. Nevertheless, preclinical studies in animal models [50] demonstrated that exogenous administration of Treg does not exacerbate immunosuppression after cerebral ischemia, and exogenous Treg administration may improve immune status after induction of ischemia. Other clinical studies [16] did not find association between Treg levels with the development of infections after stroke, so this aspect is still unclear. In our study we tried to establish the association between circulating Treg levels and development of infections during hospitalization.

We found that the presence of infections during the acute phase of stroke was associated with poor long-term outcome, as previously reported [37]. Patients with systemic infections showed higher body temperature at 24 and 48 h, suggesting that infections were early developed after ischemic stroke. Interestingly, both lower Treg levels at 48 and 72 h were independently associated to development of infections after stroke. In fact, most of the infections in our patients were detected during the early phase of stroke (first-second day), when Treg have not achieved their highest levels. In this regard, previous preclinical studies in animal models of cerebral ischemia have demonstrated that Treg exogenous administration improves immune system function, reducing the risk of spontaneous infections after MCAO [32, 49]. Our results confirm this effect described in animal models of cerebral ischemia, suggesting a possible protective role of Treg in the risk of infections, or at least not a deleterious effect.

Finally, our study has some weaknesses: First, we used CD4+, CD25+ and CD127- as membrane markers for Treg. Most authors proposed that FoxP3 is the most specific marker for Treg, but FoxP3 is an intracellular protein, so it cannot be used to isolate human Treg for functional studies or in vivo expansion for cellular therapy. We used the non-expression of CD127, which has previously demonstrated to be directly related with FoxP3 levels [21, 23]. Second, we determined Treg levels in peripheral blood but we have not demonstrated that these cells are infiltrating the brain tissue after IS. For this objective, invasive techniques would be needed to confirm the presence of Treg in ischemic lesion region. Third, Treg levels were only determined during the acute phase of IS. We did not evaluated long-term temporal profile of these cells. Finally, we have only evaluated final infarct volume (and not the brain edema), because we did not perform halftime neuroimaging studies unless the patient developed END.

## Conclusions

We found an independent association between Treg levels and good functional outcome at 3 months in IS patients. Treg levels increase after stroke and this increase was closely associated to protective effects; higher levels of these cells were associated to better functional outcome, smaller infarct volume, lower risk of END and infections during hospitalization. Treg were also correlated with IL-10 levels, supporting that this anti-inflammatory cytokine may play an important role in the beneficial effects of these cells after ischemic stroke. Therefore, Treg may be considered a potential therapeutic target in acute ischemic stroke. Finally, due to the potential relevance of Treg in stroke and other diseases, new therapeutic strategies able to increase these cells could be developed in the future.

## Data Availability

The datasets analyzed during the present study will be available from the corresponding author upon reasonable request based on the guidelines of the Ethics Committee of the Servizo Galego de Saúde.

## Abbreviations

CT: Computed tomography
END: Early Neurological deterioration
IS: Ischemic stroke
MCAO: Middle cerebral artery occlusion
MRI: Magnetic Resonance Imaging
mRS: Modified Rankin scale
NIHSS: National institute of Health Stroke Scale
rtPA: Recombinant plasminogen activator
Treg: Regulatory T cells
